# Demographic, Epidemiological and Functional Profile Models of Greek CrossFit Athletes in Relation to Shoulder Injuries: A Prospective Study

**DOI:** 10.3390/jfmk10030278

**Published:** 2025-07-18

**Authors:** Akrivi Bakaraki, George Tsirogiannis, Charalampos Matzaroglou, Konstantinos Fousekis, Sofia A. Xergia, Elias Tsepis

**Affiliations:** 1 Laboratory of Therapeutic Exercise and Sports Rehabilitation, Department of Physiotherapy, School of Health Rehabilitation Sciences, University of Patras, 26504 Patras, Greece; matzaroglou@upatras.gr (C.M.); kfousekis@upatras.gr (K.F.); sxergia@upatras.gr (S.A.X.); 2 Department of Food Science and Technology, University of Patras, 26504 Patras, Greece; gtsirogianni@upatras.gr

**Keywords:** shoulder, injury incidence, injury risk, CrossFit, functional assessment

## Abstract

**Objectives**: Shoulder injury prevalence appears to be the highest among all injuries in CrossFit (CF) athletes. Nevertheless, there is no evidence deriving from prospective studies to explain this phenomenon. The purpose of this study was to document shoulder injury incidence in CF participants over a 12-month period and prospectively investigate the risk factors associated with their demographic, epidemiological, and functional characteristics. **Methods**: The sample comprised 109 CF athletes in various levels. Participants’ data were collected during the baseline assessment, using a specially designed questionnaire, as well as active range of motion, muscle strength, muscle endurance, and sport-specific tests. Non-parametric statistical tests and inferential statistics were employed, and in addition, linear and regression models were created. Logistic regression models incorporating the study’s continuous predictors to classify injury occurrence in CF athletes were developed and evaluated using the Area Under the ROC Curve (AUC) as the performance metric. **Results**: A shoulder injury incidence rate of 0.79 per 1000 training hours was recorded. Olympic weightlifting (45%) and gymnastics (35%) exercises were associated with shoulder injury occurrence. The most frequent injury concerned rotator cuff tendons (45%), including lesions and tendinopathies, exhibiting various severity levels. None of the examined variables individually showed a statistically significant correlation with shoulder injuries. **Conclusions**: This is the first study that has investigated prospectively shoulder injuries in CrossFit, creating a realistic profile of these athletes. Despite the broad spectrum of collected data, the traditional statistical approach failed to identify shoulder injury predictors. This indicates the necessity to explore this topic using more sophisticated techniques, such as advanced machine learning approaches.

## 1. Introduction

CrossFit (CF) workouts are composed of high intensity and variety functional movements, integrating elements from weightlifting, gymnastics, and cardiovascular exercises aiming to enhance strength, stamina, flexibility, and coordination [1]. Both aerobic and anaerobic energy systems are engaged, exemplified by benchmark workouts like Isabel, which demand maximal effort and considerable energy expenditure [2]. CF athletes exhibit a force–velocity profile that is more oriented towards velocity than force, with significant differences in neuromuscular characteristics between males and females, and improvements noted with increased training frequency [3]. However, overtraining, along with the high variability of exercises and occasionally improper form, have been associated with contributing to overuse injuries [4,5].

The prevalence of injuries among CF practitioners varies significantly, with reported rates ranging from 0.2 to 18.9 injuries per 1000 h of exposure [6]. The most affected areas are the shoulders, lower back, and knees, with shoulder injuries being remarkably prevalent, accounting for up to 40.6% of all injuries [7]. These injuries are often linked to specific exercises, such as Olympic weightlifting and gymnastic movements like kipping pull-ups and ring dips [7,8]. These exercises, which are integral to CF routines, place significant stress on the shoulder joint, often leading to overloading and subsequent injuries such as partial lesions of the supraspinatus tendon and labral tears [9,10]. The repetitive and extreme positions required in CF, such as overhead movements, predispose the shoulder complex to overload and damage [11]. Additionally, improper training techniques like training with pain and inadequate warm-up routines have been shown to adversely affect injury incidence [4,5]. Recent study findings indicated an isokinetic force–power imbalance in favor of internal rotators in the shoulder of advanced CrossFitters [12]. Scapular dyskinesis, glenohumeral internal rotation deficit, and posterior shoulder stiffness, which are observed in overhead athletes’ populations, can also lead to kinematics alterations and decreased shoulder functionality [13].

In conclusion, the high prevalence of shoulder injuries in (CF) athletes could be mitigated by systematically recording predisposing factors and elucidating their contribution to injury incidence. Despite an expanding body of research, much of the current literature comprises lower-level evidence, highlighting the need for high-quality study designs [14]. The present prospective cohort study aimed to develop a representative profile of CF athletes in Greece regarding shoulder injuries. Specifically, we sought to integrate demographic, epidemiological, and functional data to investigate their association with shoulder injury occurrence over a complete training and competition cycle. Following an initial baseline assessment, athletes were monitored prospectively throughout the entire cycle to record shoulder injuries and support the modeling of potential predisposing factors.

Based on the current literature and study objectives, it was hypothesized that greater functional asymmetries, such as side-to-side deficits in strength, the range of motion, or endurance, would be associated with an increased risk of shoulder injury. Furthermore, it was anticipated that higher training volume combined with inadequate warm-up and recovery practices would correlate positively with injury incidence. Lastly, it was expected that specific performance deficits reflecting compensatory patterns related to functional limitations of the shoulder, including reduced external rotator endurance, limited mobility or lower scores in sport-specific scores, would serve as potential predictors of shoulder injury in CF athletes.

## 2. Materials and Methods

### 2.1. Participants

Four CF gyms (boxes), located in four different cities in Greece, were included in the study. They were asked to invite their athletes to participate in a shoulder injury investigation survey. The inclusion criteria were as follows: individuals of both genders, aged 18 years or older, regardless of the level of experience, while novice participants had to complete the first month of participation. Individuals were excluded from the study if they had an active injury in any area of the body which discouraged them from ordinary training routines, or they failed to participate in CF constantly for the following one year after their baseline assessment. One hundred and eleven participants met the inclusion criteria, but during the data analysis stage, two of them dropped out due to missing values. Eventually, the results from one hundred and nine healthy active CF participants entered the statistics. Detailed information about the study procedures, associated risks, and the benefits of participation was provided to all participants before reading and signing an informed consent form. The study protocol obtained ethical approval from the Ethics Committee of the University of Patras (Unique Protocol ID: 14279) and was registered in the ClinicalTrials.gov PRS database (Identifiers: NCT05909592).

### 2.2. Testing Protocol

The intention of the present study was to record CrossFitters’ profile concerning shoulder injuries and investigate shoulder injury rates and risk factors among this population. For this purpose, a unique protocol of a prospective cohort study was created. The study protocol consisted of three stages: baseline assessment, one-year monitoring and finally data analysis. During the first stage, participants were admitted to the study and underwent the baseline evaluation process. Baseline evaluation included the sequence of demographics, health status and injury history, anthropometry, flexibility, strength, muscle endurance, and functional ability assessment. Demographic data, general health history, general injuries history, and a detailed history of shoulder injuries were documented using specially designed forms. The dominant upper extremity was determined via participants’ self-report concerning the limb that demonstrated preference in performing a range of basic tasks. The entire initial assessment process took about one hour for each participant. To avoid a fatigue effect, dynamometry was performed in three trials; the functional tests were executed once and, in case of doubt, a second attempt was made for the unilateral tests and even a possible third attempt for the bilateral tests. The rest between the tests was approximately two minutes.

### 2.3. Active Range of Motion (ROM)

Shoulder ROM during flexion, external and internal rotation was evaluated qualitatively via a scale of 0–3. Normal symmetrical ROM was presented with 0, while considerable ROM restriction to both sides was presented with 3. Deficit to the dominant or non-dominant upper limb was labeled as 1 or 2, respectively. The athletes stood with their back against the wall, ensuring trunk stability, while simultaneously executing movements with both arms. The distance of each hand, at the wrist level, from the wall was measured in centimeters to decide whether symmetry or asymmetry existed. This binary approach was selected instead of goniometry, because pilot measurements inserted high uncertainty regarding the reliability of continuous variables in this position. Seated trunk rotation ROM was measured utilizing a custom-made iron rod with a laser-guided digital goniometer (HG1, HALO Medical Devices, Sydney, NSW, Australia) [15] attached on it to ensure higher accuracy of the measurements. For this examination, athletes sat astraddle on a box with ninety degrees of knee and hip flexion, feet on the ground and neutral position of their back. The rod was held by both hands at scapular spine level. Trunk rotation was slowly performed on each side and the final position in each direction was recorded in degrees.

### 2.4. Strength

The isometric strength of the shoulder external rotator muscles, internal rotator muscles and hip abductors was measured utilizing the Kinvent handheld dynamometer (K-Force Muscle, Kinvent, Montpellier, France) [16] and the Kinvent application (K-Force application, version 5.4.10, Kinvent, Montpellier, France). The dynamometer was stabilized on the wall by the examiner, two fingers below wrist level. Internal and external shoulder rotations were performed against the wall in a standing position close to the wall, with ninety-degree shoulder abduction and elbow flexion. The elbow position was marked by a tape in the wall to ensure it remained the same for both sides throughout the test. For the hip abduction, the dynamometer was stabilized against a vertical steel frame on the ground level with a strap. Participants lay on the floor with their hips at twenty degrees of abduction and zero degrees of flexion and were instructed to push out against the dynamometer as hard as they could without raising the limb from the floor. The muscle endurance of the external rotator muscles was evaluated via a protocol created in the K-Force application (version 5.4.10; previously described). Participants were asked to hold an isometric contraction from the same position for shoulder strength measurements. Three sets of thirty seconds, intercepted by three-second intervals, were performed at the target intensity of 60% of one maximum voluntary contraction (MVC). A deviation within 6% was allowed; thus, the percentage of the time maintaining at least 54% force output represented endurance performance. The output for each shoulder was a percentage of successful isometric strength production throughout the timeframe of the total 90 s, as calculated automatically by the dynamometer application.

### 2.5. Sports-Specific Tests

Functional ability was assessed using a novice sport-specific instrument, CrossFit FABS [17]. The newly introduced battery of tests consisted of six tests that are used regularly on the CF routine: air squat, shoulder mobility, wall angel, overhead squat (OHS), windmill, and Sots press. Each test was scored from 0 to 3. A total score of the tool was also calculated for each participant. Lastly, the Closed Kinetic Chain Upper Extremity Stability Test (CKCUEST) was used to evaluate shoulder dynamic stability in a familiar loading position [18]. Testing started from a push-up position with the hands placed outside two lines of tape, distanced 91.4 cm (36 inches), feet at shoulder width. They were encouraged to touch the athletic tape with the opposing hand, while alternating hands, as many times as possible within fifteen seconds. The number of repetitions achieved was recorded for each subject.

### 2.6. One-Year Monitoring and Recording Shoulder Injuries

Baseline measurements were conducted from September 2022 until February 2023. Each participant was followed for one year after entering the study. During that period, participants were contacted on a weekly basis for an update about their shoulder health. Once a new shoulder injury emerged, the athlete was examined personally by the main researcher, a physiotherapist with more than 10 years of clinical experience and expertise in sports injuries and musculoskeletal disorders. Injury identification was made through clinical examination, and the injured athlete was tracked until coming back to regular training. The criterion of a “new shoulder injury” included any new or aggravated old injury sustained during training or competition, which prevented participation in CF or urged performance modifications for tissue protection for at least one day [11,19].

### 2.7. Statistical Analysis

Variables were coded into groups according to their type (nominal, ordinal, categorical, or scalar) and their nature (for instance, ROM, strength, endurance, or specific functional tests). Descriptive statistics, including range, mean, standard deviation and standard error, as well as inferential statistics, were analyzed [20]. Non-parametric techniques, particularly the kernel density estimation method, were used to estimate the Probability Density Functions (PDFs) [21,22]. Hypothesis testing was conducted using both the t-test and the Mann–Whitney U test, along with a *p*-value correction technique to address the issue of multiple testing [23]. Linear models were employed to explore the interactions among the continuous measurements described in the previous sections and provide insights into their relationships. Finally, logistic regression models were used to link these continuous measurements with potential shoulder injuries [24]. All statistical analyses were conducted using IBM SPSS Statistics, version 29.0 (IBM Corp., Armonk, NY, USA) and Python, version 3.11 (Python Software Foundation, Wilmington, DE, USA), with the Seaborn library (version 0.12.2).

## 3. Results

### 3.1. Demographics and Injury Incidence

The study included one hundred and nine CrossFitters from four cities in Greece, ten of them (9.2%) being left-hand dominant. A complete demographic profile is illustrated in Table 1. Approximately one in five participants (19.3%) competed in CF events, while the remainder trained for health and recreation purposes. Regarding their warm-up and cool-down routines, proportions of 72.5% and 45% of the participants, respectively, declared that they were consistently adhering to both procedures in an adequate manner. Nearly half of the participants (48.6%) engaged in other sports in addition to CF. In total, 40.4% of the sample population had a particularly good fitness level before starting CF, and 35.8% started with a low prior fitness level. Notably, a fraction of four out of ten participants (N = 43, 39.4%) reported shoulder pain during CF workouts at baseline.

During the observation period, eighteen athletes (16.5%) experienced a new unilateral shoulder injury, and two had bilateral involvement, resulting in a cumulative total of twenty shoulder joint injuries. Half of the incidents (10 cases) occurred on the non-dominant side, six on the dominant side, and four injuries were bilateral. The injury incidence rate (IIR) of shoulder injuries in this sample of CF athletes was 0.79 per 1000 training hours.

Six out of the twenty referred shoulder injuries were new, while the remaining fourteen were an exacerbation of an old injury. Four injuries were acute, whereas the remaining sixteen demonstrated gradual onset as overuse injuries. The occurrence of 45% of the new shoulder injuries was associated with Olympic weightlifting exercises, 35% with gymnastics, and equal percentages of 10% with a combination of Olympic weightlifting and gymnastics and with free weights exercises solely.

In terms of the tissue involved, the most frequent injury (45%) concerned rotator cuff (RC) tendons, including lesions and tendinopathies. The second most prevalent injury, accounting for 40%, was identified as a concomitant injury to the rotator cuff and the bicep tendon. Labral tears and injuries involving both the rotator cuff and the acromioclavicular joint were noted, representing 10% and 5% of cases, respectively. The injury severity ranged from the modification of a single-day workout to the alteration or total cessation of practice for up to 108 days. Only five injured participants (25%) managed to return to sport without pain, while twelve (60%) returned to full practice with pain symptoms, and the remaining three (15%) failed to return to their previous level of practice routines during the observation period. The pain, shoulder disability, and days out of practice are shown in Table 2.

### 3.2. Exploratory Data Analysis and Descriptive Statistics

During the first stage of statistical analysis, potential factors that could influence injury rates were investigated to determine whether further grouping based on variables such as gender, age, experience, or competition level was necessary. Apart from statistical analysis, PDFs of the binary variables were used to illustrate the degree their distributions overly.

#### 3.2.1. Demographic Data

**Figure 1:**
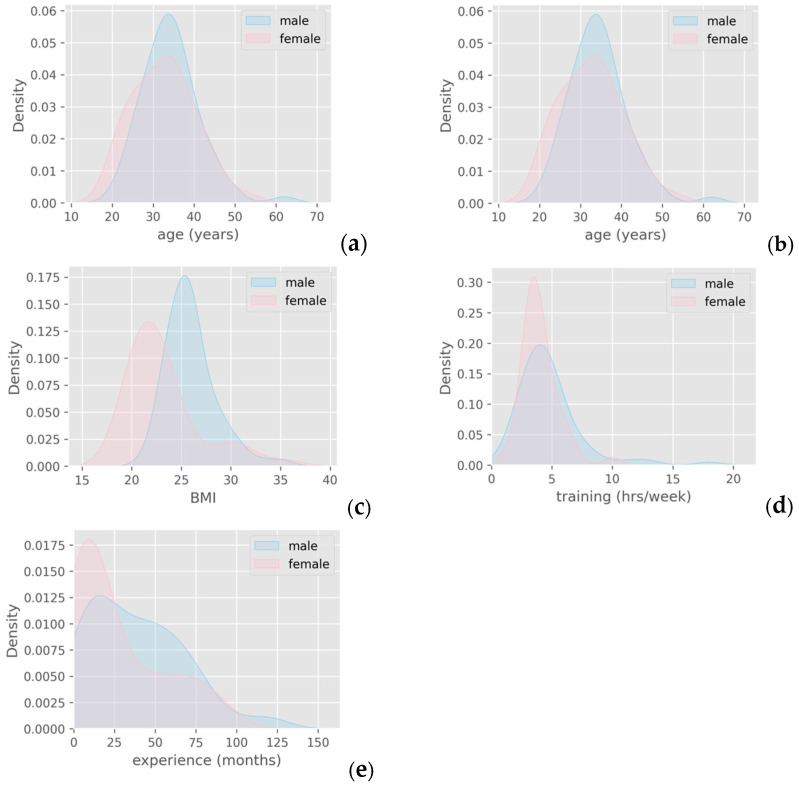
Descriptive statistics and PDFs of male and female athletes (**a**) regarding (**b**) age, (**c**) BMI, (**d**) training volume, and (**e**) CF experience.

Male athletes represented 61.5% of the sample. Regarding gender profile, PDFs of male and female athletes showed considerable overlapping in age and training volume, visually verifying their non-statistically significant difference. The only noticeable observation, however non-significant, was that male athletes exhibited higher BMI values and were slightly more experienced (Figure 1).

**Figure 2:**
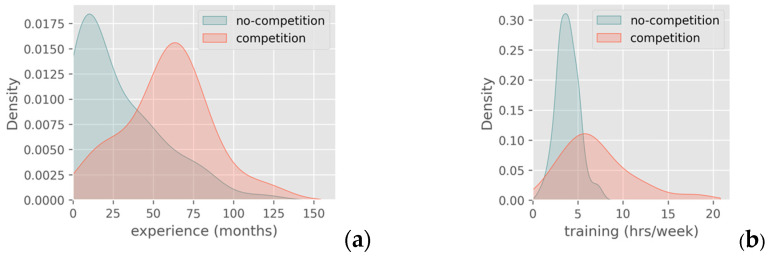
PDFs of competitive and non-competitive athletes regarding (**a**) experience and (**b**) training volume.

Competitive athletes exhibited very similar age and BMI distributions to non-competitors. However, their PDFs for experience and weekly training hours were shifted slightly toward higher values (Figure 2). Based on the CF age categorization threshold of 40 years [25,26], it was noticed that 83.5% of the sample was classified as younger athletes. The BMI was modestly higher in the older subgroup, while training volume and experience showed no substantial variation between age groups.

**Figure 3:**
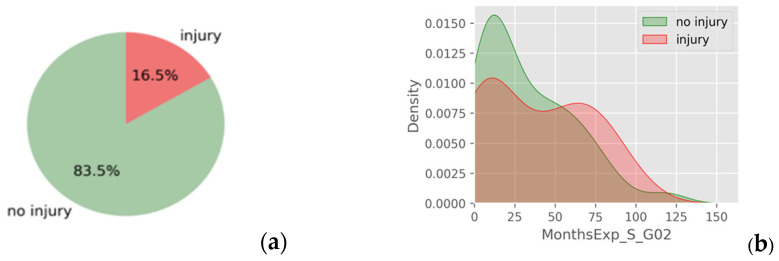
(**a**) Sample’s percentage of injured and non-injured athletes and (**b**) an indicative example of overlapping in PDF of injured and non-injured athletes regarding CF experience.

When comparing athletes who sustained a new shoulder injury and those who did not, overlapping PDFs were also observed across age, BMI, experience, and training volume (Figure 3), suggesting limited group-level differentiation in these variables.

#### 3.2.2. Range of Motion, Strength and Sports-Specific Tests

**Figure 4:**
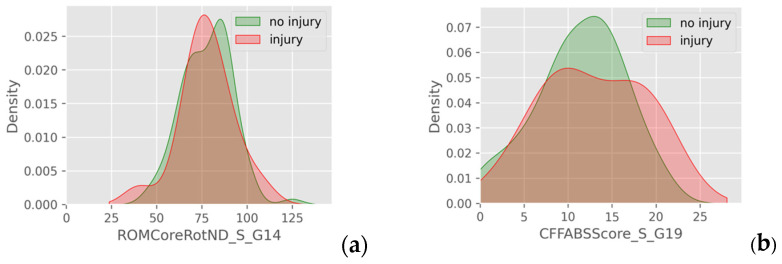
PDFs of injured and non-injured athletes regarding (**a**) seated trunk rotation towards NDUE’s side and (**b**) CF FABS total score.

Overlayed probability density plots showed substantial overlap in continuous variables between injured and non-injured athletes. In particular, variables such as seated trunk rotation ROM toward the side of the non-dominant upper extremity (NDUE), hip abduction strength in both sides and CKCUEST scores revealed nearly identical distributions across groups. A minimally reduced overlap was noted in CF FABS total scores, hip abduction strength deficit, non-dominant shoulder external rotation endurance, and shoulder external rotation endurance deficit (Figure 4). Notwithstanding, even these differences were subtle and localized near the distribution peaks, with their overall spread remaining similar. This consistent pattern of overlap indicates minimal distinction between the two groups in these functional measurements.

#### 3.2.3. Ordinal Measurements and Data

**Figure 5:**
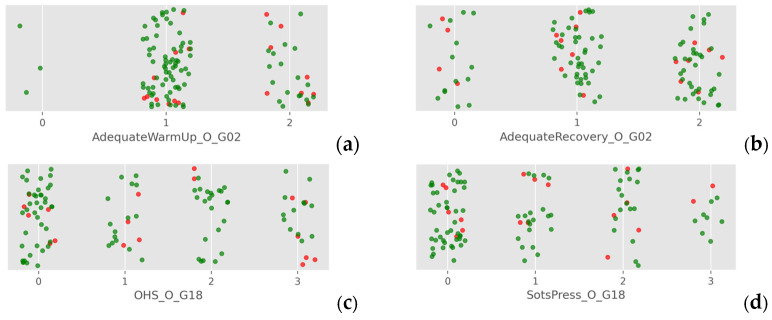
Injured (red dots) versus non-injured (green dots) athletes regarding ordinal data of (**a**) warm-up [0 = never, 1 = sometimes, 2 = every time], (**b**) recovery [0 = never, 1 = sometimes, 2 = every time], (**c**) OHS [0 = pain/inability, 1 = poor performance, 2 = average performance, 3 = excellent performance], and (**d**) Sots press test [0 = pain/inability, 1 = poor performance, 2 = average performance, 3 = excellent performance].

A visual inspection of ordinal data (e.g., warm-up routines, recovery practices, prior fitness level, injury history, and individual CF FABS test scores) revealed no distinguishable patterns between injured and non-injured athletes. Injured and non-injured individuals were intermixed across all ordinal categories, indicating that no single measure effectively differentiated the two groups. This technique prevented data collapse while preserving the categorical structure, allowing for a clearer distinction between injured and non-injured athletes (Figure 5).

#### 3.2.4. Nominal Data

**Figure 6:**
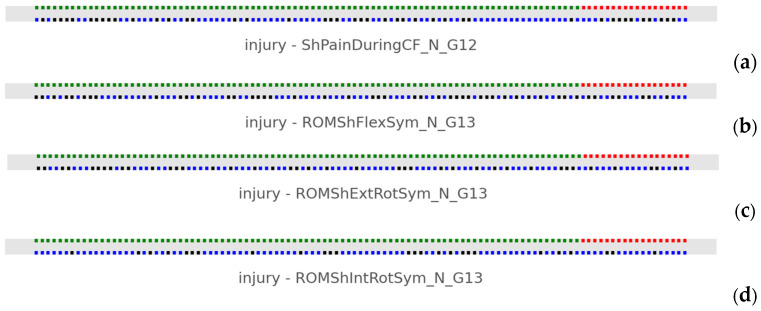
Examples of visual verification of binary data intermixture. Injured versus non-injured athletes regarding (**a**) shoulder pain during CF, (**b**) symmetry of flexion ROM, (**c**) external rotation, and (**d**) internal rotation ROM between shoulders.

A typical example of binary categorical data interaction is visually represented in Figure 6. Injured and non-injured athletes were plotted against the binary response of each categorical variable. We attempted to locate any systematic pattern that could potentially connect the two colored lines, for example, a pattern that blue or black dots favored any of the red or green athletes. It was observed that this pattern was absent in all subplots concerning gender, UE dominance, competitiveness, other parallel activities, and history of general injury. In particular, there was a significant intermixture of binary data across all groups.

### 3.3. Inferential Statistics

Chi-squared (χ^2^) tests were used to compare injury prevalence across categorical subgroups. The findings indicated that a proportion of 19.4% of the male population exhibited a shoulder injury in contrast to the corresponding 11.9% of the female population (χ^2^ = 1.053, *p* = 0.305). Moreover, 23.8% of the competitive athletes sustained injuries, as opposed to 14.8% of the non-competitive athletes (χ^2^ = 0.219, *p* = 0.640). Nearly identical proportions of younger and older participants (16.5% and 16.7%, respectively) displayed shoulder injuries throughout the observational period (χ^2^ < 0.001, *p* = 0.985). The *p*-values for these statistical analyses were notably elevated, suggesting a lack of statistical significance, and therefore, no further pairwise tests were conducted.

Independent *t*-tests assess continuous variables between injured and non-injured groups, applying a Bonferroni-adjusted alpha significance level of 0.0031 to account for 16 multiple tests [23] (Table 3). Likewise, ordinal variables were analyzed using the Mann–Whitney U test with a corrected threshold of α = 0.0029 for 17 multiple comparisons (Table 4). Most *p*-values exceeded these conservative thresholds, indicating no significant group differences.

The low *p*-values of the hip abductors’ strength deficit, shoulder external rotators’ endurance deficit, warm-up practice, and history of body injuries noted a difference between the tested variables. These may point to a better attitude towards warm-up in CF athletes and a possible relationship between specific deficits or general injury history and shoulder injury incidence. However, after Bonferroni correction due to the high number of comparisons, this difference was not considered statistically significant. These findings may inform future hypothesis generation and model refinement.

### 3.4. Understanding the Interactions of the Epidemiological Characteristics and the Functional Tests

**Figure 7:**
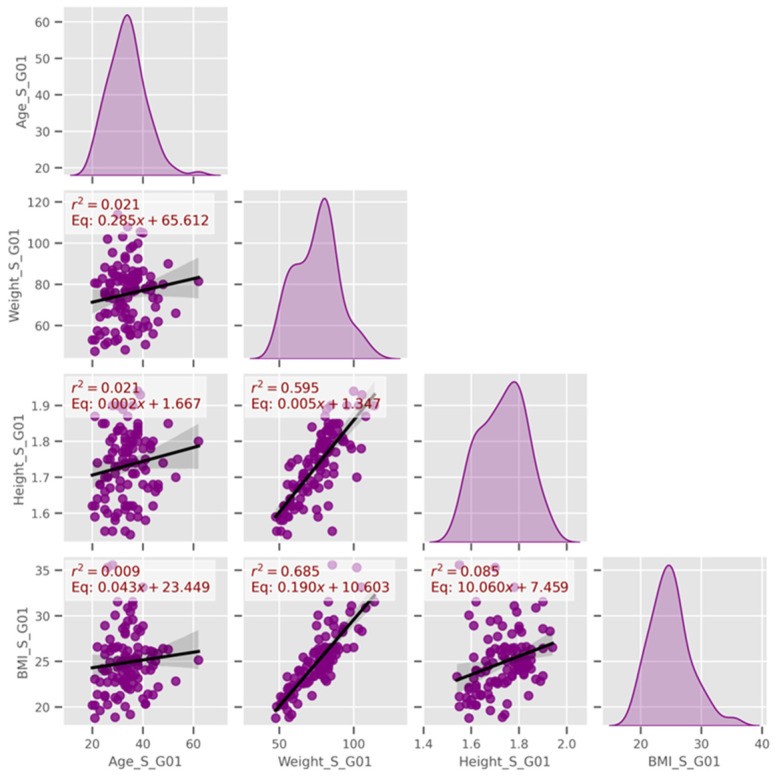
Linear models among age and weight, height, and BMI (data group 1).

Ordinary least square (OLS) linear regression models were used to explore relationships among continuous variables within each coding group. As shown in Figure 7, height and weight were strongly correlated, suggesting that a lean body type may be typical among CF participants. Conversely, age and weight showed little association (low R^2^ values), which likely reflects the sport’s high energy demands and consistent training habits. A moderate positive correlation between experience and weekly training hours suggests that more experienced athletes tend to maintain higher training volumes.

**Figure 8:**
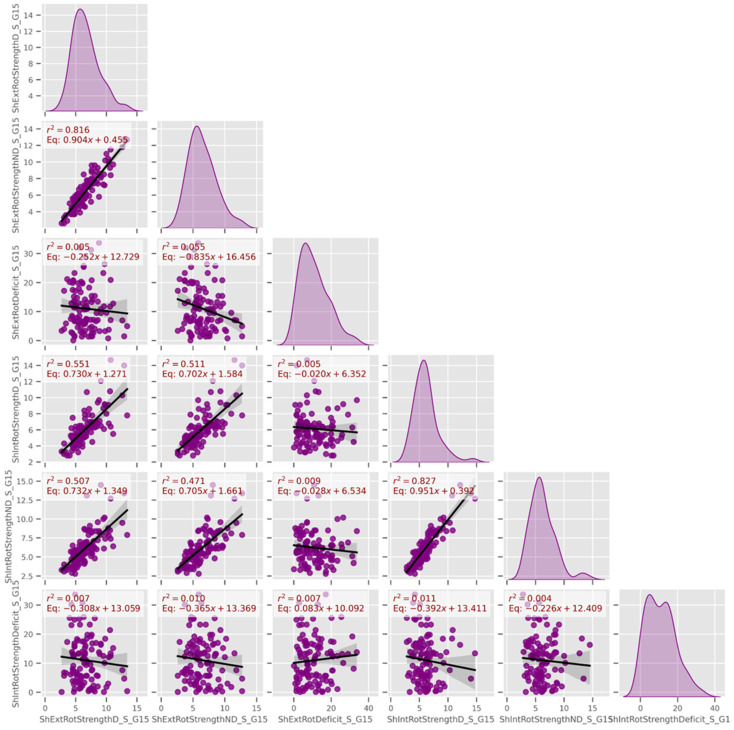
Linear models among dominant and non-dominant shoulder’s external and internal rotation strength. Continuous data of external and internal rotators strength and deficits (Data group 15).

In Figure 8, dominant and non-dominant shoulder external rotator strength, as well as internal rotator strength, displayed very high R^2^ values, indicating strong bilateral symmetry. This supports the idea that CF training promotes balanced shoulder development, which is a potential prerequisite for effective performance. A moderate correlation (R^2^ = 0.4) was also found in seated trunk rotation ROM between sides, further reinforcing this bilateral symmetry pattern.

**Figure 9:**
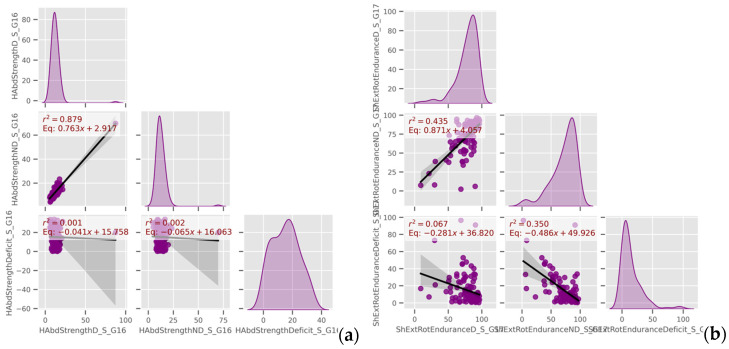
Linear models among (**a**) hip abductor strength measurements and (**b**) shoulder external rotator endurance measurements (data group 16 and 17, respectively).

Similarly, Figure 9 shows consistent bilateral correlations for hip abduction strength and shoulder external rotator endurance. These results align with the demands of CF, which emphasizes multi-planar, high-intensity movements and may encourage symmetrical muscular development over time.

### 3.5. Modeling and Predicting Shoulder Injuries

Models of the CF athletes’ injuries that utilize the continuous (i.e., scalar) variables of this study were created. Logistic regression models [24] that combine the continuous variables (i.e., predictors) with the binary outcome of injury/no-injury (i.e., response variable) are presented. The model’s performance metric utilized the Area Under the Curve (AUC) of the Receiver Operating Characteristic (ROC) curve, which quantifies the overall ability of a binary classifier to discriminate between the two classes across all possible classification thresholds [24]. An AUC value of 1.0 indicates perfect classification, while a value of 0.5 suggests no discriminative ability, equivalent to random guessing. Higher AUC values reflect better model performance. The ROC curve plots the true positive rate (TPR or sensitivity: measures how well a model identifies actual positive cases, and quantifies how many the model correctly caught out of all the real positives/injuries) against the false positive rate (FPR or specificity: measures how often a model incorrectly labels negative cases as positive, and quantifies how many the model mistakenly flagged as positive out of all the real negatives/no injuries), showing the trade-off between sensitivity and specificity. The AUC is widely used because it is independent of class distribution and provides a single, interpretable measure.

**Figure 10:**
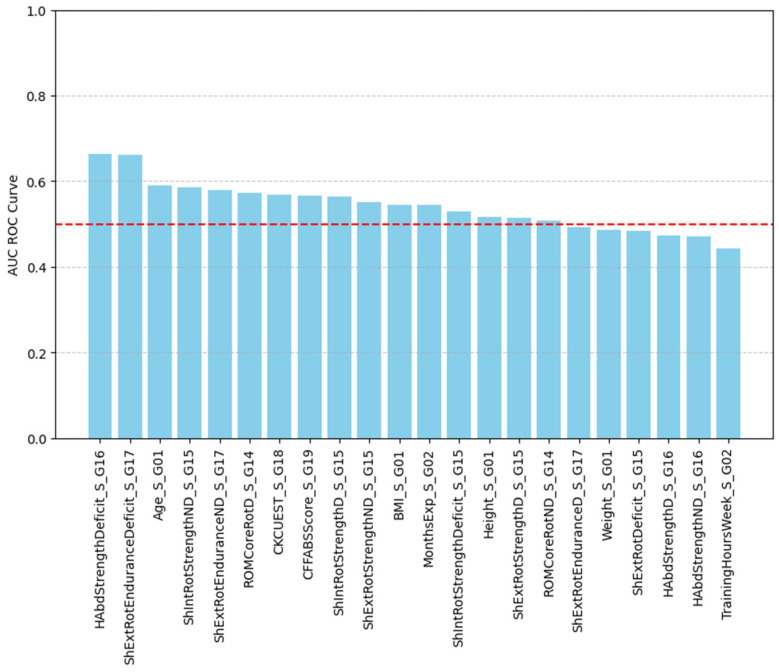
The AUC performance of the logistic regression for all the continuous variables. Red dashed line represents the level of complete randomness (coin flip).

In Figure 10, the AUC performance of the logistic regression models is presented, computed for all continuous variables analyzed in this study. As expected, given the large *p*-values from the corresponding t-tests and the substantial overlap of the probability density functions, the model’s performance is generally low, with most of the AUC values close to 0.5. This provides strong evidence that individual variables possess limited or negligible predictive capability for shoulder injuries.

**Figure 11:**
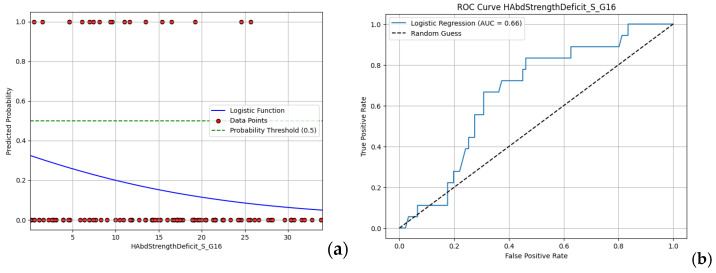
(**a**) Logistic regression model and (**b**) ROC curve for hip abduction strength deficit.

**Figure 12:**
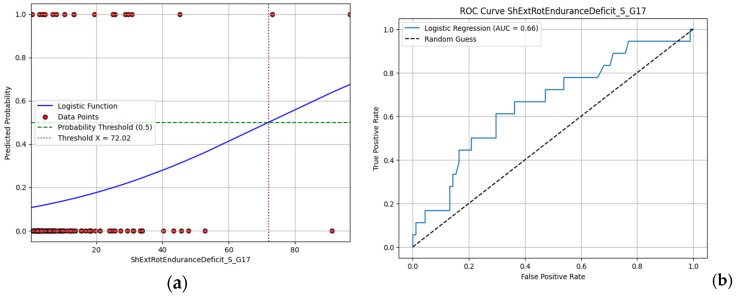
(**a**) Logistic regression model and (**b**) ROC curve for shoulder external rotation endurance deficit.

Focusing on moderate performance models, with AUC values around 0.66, hip abduction strength deficit and shoulder external rotation endurance deficit are presented in Figure 11 and Figure 12. Higher values of shoulder external rotation endurance deficit appear to favor injury occurrence (i.e., >70) with a steady gaining ROC curve for the whole range of the values (with respect to random choice). On the other hand, the ROC curve of hip abduction strength deficit appears to be problematic at lower values (i.e., < 0.2), indicating that the model fails to accurately capture this range. Overall, even the best-performing models offer limited predictive value, reinforcing the need for more complex, multifactorial approaches in future analyses.

## 4. Discussion

Numerous epidemiological investigations have examined CF injuries in recent years, revealing a significant prevalence of shoulder injuries. To the fullest extent of the researchers’ knowledge in the current study, this represents the inaugural prospective investigation into the demographic, epidemiological, and functional characteristics of shoulder injuries among CF athletes. The observed IIR for shoulder injuries was calculated to be 0.79 per 1000 training hours, which is considerably lower than the results of prior, isolated retrospective epidemiological research on shoulder injuries that documented an IIR of 1.18 [27]. The results of the present study resembled another retrospective investigation that solicited participants to recount their injuries that transpired within the previous six months, ultimately counting an IIR of 0.51 for shoulder injuries [28]. It was observed that there was high variability in injury reports among previous retrospective studies, nonetheless. This disparity may be attributed to the self-reported nature of data collection in previous studies, which relied on participants’ evocation of their past injury experiences, contrary to the current study, which used a prospective methodology. In order to enhance the precision of the results, participants were tracked by researchers during a year of training and competitions, with the objective of documenting any shoulder injuries that occurred during this period, including the nature, severity, and impact on the athlete’s participation in CF.

An additional innovative aspect of the present research was the implementation of the CF FABS, a sport-specific assessment tool utilized to evaluate the functional capabilities of CF athletes. The only prior assessment tool employed for the CF athlete population was the Functional Movement Screen (FMS) [29]. However, this is a widely used tool for the assessment of functionality, while CF FABS is specific for the sport. The aggregate scores obtained from both assessment tools did not reveal statistically significant correlations with the incidence of shoulder injuries. A noteworthy finding was that CrossFitters exhibited remarkably higher symmetry on the FMS in comparison to other athletic populations, particularly in the shoulder mobility test [30]. The present study’s results corroborated those suggestions by demonstrating notable symmetry in ROM, strength, and endurance measured parameters. These findings regarding highly symmetrical performance may account for the absence of a statistically significant correlation between side-to-side differences and shoulder injury incidence.

CF has been characterized as a strenuous sport, pushing participants to engage at the upper limit of their physical capacities [31]. The toughness of the sport in addition to the sample’s average time of attendance, which was approximately three years, may indicate that athletes were used to participating under hard circumstances. It appears that substantial pain tolerance has been evidenced, indicated by the finding that nearly 40% of our sample were practicing regularly despite experiencing pain in their shoulders. This element may serve as a contributory factor to the observed low incidence rate of shoulder injuries.

As a new injury was delineated, any sensation of pain or discomfort resulting in the cessation or alteration of the training regime for one day or more was noted. The criteria for defining a newly reported injury were consistent with established definitions in the literature [27,29]. Prior prospective investigations on the incidence of CF injuries and their corresponding risk factors tracked their cohorts for durations of either 8 weeks [32] or 12 weeks [19,29], which were considerably shorter time frames than the 12-month period of the current study.

Previous research has indicated that age and sex represent significant non-modifiable factors correlated with the incidence of injuries, with older individuals and male participants demonstrating a heightened susceptibility to such injuries [5,6]. Moreover, an elevated BMI has been associated with a higher risk for injury, alongside a prior history of injuries, which predisposes individuals to a greater likelihood of sustaining subsequent injuries, particularly in the same anatomical region [6]. A discrepancy was observed among studies regarding competitiveness. One particular study found a higher injury incidence among competitive individuals [28], a different one reported a 2.64-fold greater probability for injury in non-competitors [33], while another study showed no statistically significant differences between the groups [34]. One possible explanation for these contradictory results, apart from the diverse methodological approaches of the studies, is the fact that competitive athletes are usually exposed to a higher load by pushing their limits further, which may be counteracted by them being more physically aware and technically efficient. Concerning male gender, older age, and prior injury history, a contemporary systematic review on the vulnerability to musculoskeletal injuries among CF participants resulted in incongruent evidence [35]. This is in line with the current study, which demonstrated no statistically significant association of any of those variables with the incidence of shoulder injuries.

RC injuries constituted the predominant tissue involvement among the shoulder injuries that occurred, manifesting either in isolation or in conjunction with other shoulder injuries. This result was in accordance with the outcomes of a magnetic resonance imaging study, which indicated elevated percentages of partial lesions in the supraspinatus and subscapularis tendons within a sample of CF participants evaluated [9]. In terms of exercises associated with an increased risk of shoulder injury, a recent systematic review identified several gymnastics exercises, namely ring dips, ring muscle-ups, and kipping movements, as significantly prevalent [7]. On the contrary, the present study recognized Olympic weightlifting exercises as the most strongly related to shoulder injuries, followed by gymnastics activities. Similarly, Summitt et al. reported that overhead press and snatch variations were the ones most frequently leading to shoulder injuries, ahead of the gymnastics exercises [27]. In summary, both types of exercises that load the shoulder in an overhead position predispose athletes to overuse tendinopathies and partial or complete thickness tears [14].

Nevertheless, the comparisons drawn between the present study and prior retrospective or prospective studies with a short observation period must be critically scrutinized. Athletes with extensive experience in CF may exhibit a higher incidence of injuries compared to those who are relatively new to the sport, reflecting chronically accumulated microtrauma. The fact that the majority of the findings come from retrospective studies based on electronic questionnaires, inherently subject to bias, weakens the existing evidence on the topic of CF injuries. Furthermore, the preponderance of the studies focuses on all forms of CF injuries, whereas the present study exclusively examined shoulder injuries. Consequently, the validity of comparisons may be debatable.

The principal objective of this investigation was to discern potential variables that may correlate with shoulder injuries among CF athletes. To accomplish this, the study’s methods were meticulously crafted to gather a comprehensive array of measurements and data. This extensive data framework affords the opportunity for an in-depth analysis intended to elucidate highly correlated variables, which may facilitate the exclusion of certain factors in subsequent research and reveal novel interactions that could enhance athletic training practices. Nevertheless, one plausible interpretation of the aforementioned statistical findings is that no single parameter in isolation, regardless being a demographic characteristic, an epidemiological variable, or a functional assessment, proved adequate to establish a link with shoulder injuries. Further inquiry may elucidate the interrelationships among variables that could contribute to the prevalence of shoulder injuries.

The principal strength of the current investigation lies in the 12-month prospective nature of the observational period, involving a cohort of 109 CF athletes. The methodological approach encompassed a direct baseline assessment of the athletes and a second assessment upon the occurrence of new injuries. The classification of the documented shoulder injuries was undertaken by a single examiner, the principal investigator who is a senior physiotherapist. A comprehensive array of measurements was collected to guarantee that all pertinent dimensions of the subject matter were thoroughly examined. Nevertheless, potential limitations were recognized. The extended duration of the study, in addition to the spread of the sample over four different cities, rendered it challenging to ensure compliance within the cohort. It is possible that some injuries went unreported to the investigators, notwithstanding the frequent updates provided to the sample. The number of the 20 new shoulder injuries might appear low, having reduced statistical power and hindered the detection of significant associations. However, the prospective monitoring of a greater sample of CF athletes is very challenging. Another possible confounding factor is pain-response bias; notably, a large proportion of the sample reported training through shoulder pain, which may have masked emerging injury patterns or delayed proper diagnosis. Additionally, the possibility that baseline values might change throughout the 12-month monitoring period introduces a degree of uncertainty to the investigation of their connection to new injuries, although this is a standard flaw of all relevant studies that use baseline tests. Repeated testing throughout the season could ameliorate this limitation. Furthermore, implementing reliable instruments to systematically track the progression of training volume and intensity in CF, as well as incorporating some potentially influential variables that were not investigated in the present study, for instance, exercise technique and fatigue management, will enhance knowledge on the topic of shoulder injury risks.

## 5. Conclusions

This prospective cohort study investigated the profile of CF athletes and the prevalence of shoulder injuries among them. Potential injury risks related to demographic, epidemiological, and mainly functional parameters associated with this specific injury category were explored. The sample demonstrated symmetrical functional characteristics, and an IIR of 0.79 per 1000 training hours for shoulder injuries was documented. Most of the cases involved RC injuries and were predominantly associated with Olympic weightlifting exercises. The severity of the cases demonstrated a broad spectrum, with all injuries being managed conservatively. Notably, a substantial proportion of athletes returned to sport despite ongoing shoulder pain. Hypothesis testing, with the common approach for epidemiological studies, indicated that none of the examined variables had a statistically significant association with the incidence of shoulder injuries.

Despite the abundance of the gathered data, there was no significant interaction between any of them and the occurrence of shoulder injuries using traditional hypothesis testing. This lack of significant findings, however, does not imply the absence of underlying risk factors. On the contrary, it highlights the need to examine such a multifaceted phenomenon of complex interactions between intrinsic and extrinsic risk factors and triggering events [36] under a novel approach. Artificial Intelligence (AI) and machine learning (ML) techniques have the potential to identify injury prone athletes and discern the most salient injury risk factors [37]. ML approaches, such as decision trees, support vector machines, or ensemble models, can accommodate multicollinearity, interaction effects, and nonlinearity more effectively than classical regression models. The results of the current study will be further explored utilizing ML algorithms to explore whether more sensitive or complex models can uncover injury-prone profiles that traditional methods overlooked.

## Figures and Tables

**Table 1:** Descriptive statistics for baseline measurements.

Variable	Mean	Std. Deviation	Std. Error Mean	Range
Age (years)	33.73	7.31	0.70	20–62
Weight (kg)	75.24	14.57	1.40	47.50–113.90
Height (meters)	1.73	0.10	0.01	1.54–1.94
BMI (kg/m^2^)	24.89	3.34	0.32	18.79–35.59
Experience (months)	34.10	29.16	2.79	1–120
Training Volume (hours/week)	4.48	2.36	0.23	1–18
Seated Trunk Rotation ROM Toward D * Side (Degrees)	73.32	14.07	1.35	40–105
Seated Trunk Rotation ROM Toward ND ** Side (Degrees)	77.92	13.87	1.33	40–125
Shoulder External Rotation Strength D (kg)	6.66	2.16	0.21	2.70–13.40
Shoulder External Rotation Strength ND (kg)	6.48	2.16	0.21	2.60–12.70
Shoulder External Rotation Strength Deficit (Side-to-Side Difference%)	11.05	7.67	0.74	0.10–33.70
Shoulder Internal Rotation Strength D (kg)	6.13	2.12	0.20	2.80–14.70
Shoulder Internal Rotation Strength ND (kg)	6.22	2.22	0.21	2.80–14.50
Shoulder Internal Rotation Strength Deficit (Side-to-Side Difference%)	11.01	7.80	0.75	0.10–33.70
Hip Abduction Strength D (kg)	12.77	7.99	0.77	5.6–87.30
Hip Abduction Strength ND (kg)	12.66	6.50	0.62	4.30–69.80
Hip Abduction Strength Deficit (Side-to-Side Difference%)	15.24	9.25	0.89	0.10–34
Shoulder External Rotation Endurance D (%)	78.37	15.98	1.53	9.10–96.50
Shoulder External Rotation Endurance ND (%)	72.31	21.11	2.02	2.40–96.90
Shoulder External Rotation Endurance Deficit (Side-to-Side Difference%)	14.77	17.35	1.66	0.40–96.60
CKCUEST Score (Non-Dimensional)	24.39	5.27	0.51	13–37
CF FABS Score (Non-Dimensional)	11.61	5.16	0.49	0–22

* D: dominant upper extremity (UE), ND **: non-dominant UE.

**Table 2:** Descriptive statistics of the new referred shoulder injuries scale data.

Variable	Mean	Std. Deviation	Std. Error Mean	Range
VAS Pain Score	5.15	1.69	0.38	3.0–9.0
SDQ Score (%)	38.13	16.83	3.76	12.50–87.50
Days Out	13.75	28.04	6.27	0–108

**Table 3:** Comparisons between the continuous data of functional measurements.

Variable	T-Statistic	*p*-Value
Seated Trunk Rotation ROM Toward D Side (degrees)	−0.8749	0.384
Seated Trunk Rotation ROM Toward ND Side (degrees)	−0.1206	0.904
Shoulder External Rotation Strength D (kilograms)	0.4344	0.665
Shoulder External Rotation Strength ND (kilograms)	0.8175	0.416
Shoulder External Rotation Strength Deficit (side-to-side difference%)	−0.2284	0.820
Shoulder Internal Rotation Strength D (kilograms)	1.8113	0.073
Shoulder Internal Rotation Strength ND (kilograms)	1.4597	0.147
Shoulder Internal Rotation Strength Deficit (side-to-side difference%)	−0.3061	0.760
Hip Abduction Strength D (kilograms)	−0.2777	0.782
Hip Abduction Strength ND (kilograms)	−0.2952	0.768
Hip Abduction Strength Deficit (side-to-side difference%)	−2.2162	0.029 *
Shoulder External Rotation Endurance D (%)	−0.2963	0.768
Shoulder External Rotation Endurance ND (%)	−1.6982	0.092
Shoulder External Rotation Endurance Deficit (side-to-side difference%)	2.6192	0.010 *
CKCUEST (nd)	−0.7786	0.438
CF FABS Score (nd)	1.0984	0.275

* Statistically significant individually, but non-significant after Bonferroni adjustment.

**Table 4:** Comparisons between the ordinal epidemiological data and sport-specific testing measurements.

	Variable	T-Statistic	*p*-Value
Epidemiological characteristics	Adequate Warm-Up	611.0	0.029 **
	Adequate Recovery	850.5	0.784
	Prior Fitness Level	903.5	0.464
	DUE * Injured Areas	663.5	0.153
	NDUE * Injured Areas	636.0	0.082
	DLE * Injured Areas	776.5	0.691
	NDLE * Injured Areas	742.0	0.442
	Core Injured Areas	663.5	0.153
	All Injured Areas	509.5	0.0097 **
CF FABS individual tests	Squat	695.5	0.289
	Shoulder Rotation D	825.0	0.962
	Shoulder Rotation ND	680.5	0.243
	Wall Angel	932.0	0.326
	OHS	688.0	0.262
	Windmill D	812.0	0.956
	Windmill ND	651.0	0.152
	Sots Press	684.0	0.241

* DUE: dominant upper extremity, NDUE: non-dominant upper extremity, DLE: dominant lower extremity, NDLE: non-dominant lower extremity. ** Statistically significant individually, but non-significant after Bonferroni adjustment.

## Data Availability

Date will be available uppon request.
